# Dynamic Approximate Entropy Electroanatomic Maps Detect Rotors in a Simulated Atrial Fibrillation Model

**DOI:** 10.1371/journal.pone.0114577

**Published:** 2014-12-09

**Authors:** Juan P. Ugarte, Andrés Orozco-Duque, Catalina Tobón, Vaclav Kremen, Daniel Novak, Javier Saiz, Tobias Oesterlein, Clauss Schmitt, Armin Luik, John Bustamante

**Affiliations:** 1 Centro de Bioingeniería, Universidad Pontificia Bolivariana, Medellín, Colombia; 2 GI²B, Instituto Tecnológico Metropolitano, Medellín, Colombia; 3 Czech Institute of Informatics, Robotics and Cybernetics, Czech Technical University in Prague, Prague, Czech Republic; 4 Department of Cybernetics, Faculty of Electrical Engineering, Czech Technical University in Prague, Prague, Czech Republic; 5 I3BH, Universitat Politècnica de València, Valencia, Spain; 6 Institute of Biomedical Engineering, Karlsruhe Institute of Technology (KIT), Karlsruhe, Germany; 7 Medizinische Klinik IV, Staedtisches Klinikum Karlsruhe, Karlsruhe, Germany; Gent University, Belgium

## Abstract

There is evidence that rotors could be drivers that maintain atrial fibrillation. Complex fractionated atrial electrograms have been located in rotor tip areas. However, the concept of electrogram fractionation, defined using time intervals, is still controversial as a tool for locating target sites for ablation. We hypothesize that the fractionation phenomenon is better described using non-linear dynamic measures, such as approximate entropy, and that this tool could be used for locating the rotor tip. The aim of this work has been to determine the relationship between approximate entropy and fractionated electrograms, and to develop a new tool for rotor mapping based on fractionation levels. Two episodes of chronic atrial fibrillation were simulated in a 3D human atrial model, in which rotors were observed. Dynamic approximate entropy maps were calculated using unipolar electrogram signals generated over the whole surface of the 3D atrial model. In addition, we optimized the approximate entropy calculation using two real multi-center databases of fractionated electrogram signals, labeled in 4 levels of fractionation. We found that the values of approximate entropy and the levels of fractionation are positively correlated. This allows the dynamic approximate entropy maps to localize the tips from stable and meandering rotors. Furthermore, we assessed the optimized approximate entropy using bipolar electrograms generated over a vicinity enclosing a rotor, achieving rotor detection. Our results suggest that high approximate entropy values are able to detect a high level of fractionation and to locate rotor tips in simulated atrial fibrillation episodes. We suggest that dynamic approximate entropy maps could become a tool for atrial fibrillation rotor mapping.

## Introduction

Catheter ablation based on mapping procedures has revolutionized the treatment of atrial fibrillation (AF). Electroanatomical mapping for guided AF ablation provides a 3D reconstruction of the cardiac chambers together with electrical information obtained from electrograms (EGM). Mappings of activation waves, voltage, dominant frequency and complex fractionated atrial electrograms (CFAE) are used to localize target sites for ablation. However, there are limitations with these techniques, which depend heavily on the expertise of the electrophysiologist [1].

CFAE mapping is still a debated technique [2]. CFAE is a physiopathological concept that was introduced by Nademanee [3]. However, this concept is broadly and unclearly defined, and involves inherent subjectivity [4]. This can lead to incorrect detection of target sites for ablation, mistaking EGM that are fractionated and functional in nature [5]. It also makes studies difficult to compare. While the concept of CFAE has made a relevant contribution to the study of AF, it may fail to describe the wide range of EGM fractionation that occurs in specific cases. In addition, inconsistent results have been found using the CFAE concept. Taking this into account, recent studies have helped to understand the concept of CFAE as a nonlinear phenomenon for quantifying various CFAE patterns, without using cycle length criteria [6]–[8].

Studies have shown that sites representing AF substrates are characterized by a high degree of disorganization in EGM [9], and, accordingly, methods for EGM signal processing are being designed to quantify the degree of fractionation of EGM [7], [8]. The relationship between CFAE and the rotor tip has been reported in recent studies [7], [10]–[13], but automatic rotor mapping methods have not been fully established.

The rotor hypothesis described in [14] proposes that in a significant number of patients, a rotor or a small number of rotors are drivers which maintain the arrhythmia. A rotor is a vortex of a spiral wave rotating around an unexcitable core. Narayan et al have provided evidence that human AF can be sustained by localized rotors [15], [16].

Based on the rotor hypothesis, which surmises that sustained AF depends on uninterrupted periodic activity of the discrete reentrant site, and on evidence that a high degree of irregularity is present in EGM signals at the rotor tip, we hypothesized that 1) the level of fractionation on EGM can be measured using a non-linear index such as Approximate Entropy (ApEn), and 2) high levels of ApEn can be used to localize the rotor tip.

## Materials and Methods

**Figure 1:**
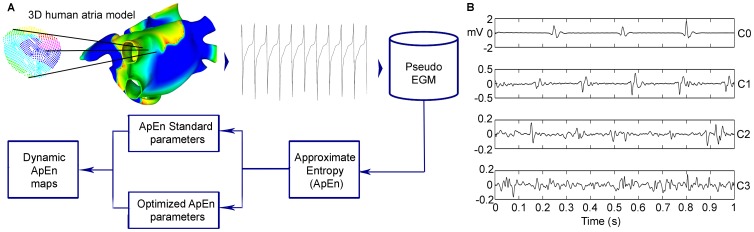
Experimental setup. A: Simulated episode of chronic AF in a 3D model of human atria. A local activation time map was constructed as an alternative method for detecting rotors. A pseudo-EGM signal was calculated from the 3D model. ApEn was calculated in pseudo-EGM signals recorded over the whole atrial surface. ApEn maps were constructed in order to observe the relation between ApEn values and rotor locations. B: Examples of EGM signals of DB-CZ-DE. Representatives from the four levels of complexity proposed for the purposes of the study are shown from C0 to C3. These signals were used for ApEn parameter optimization.

We present dynamic ApEn maps as a new tool for rotor detection. ApEn values for each EGM recorded from the atrial surface are used to construct a color map. We assess the method in a 3D computational model of human atria. Fig. 1A schematically illustrates the methodology used in this work, as follows. First, two AF episodes were simulated: a chronic AF episode in which two stable rotors were found, and a chronic AF episode in which a meandering rotor was found. Second, ApEn measurements, using standard parameters, are calculated over virtual EGM recorded from the 3D model to construct dynamic ApEn maps. Third, the ApEn parameters were optimized using real measured bipolar EGM from multi-center databases. Fourth, dynamic ApEn maps were generated using optimized parameters. Next, the rotors tips are identified by analyzing local activation time maps and dynamic ApEn maps. The details of each step are presented in the following sections.

### Chronic atrial fibrillation model

A realistic 3D model of human atria including the main anatomical structures, fiber orientation, electrophysiological and conduction heterogeneity and anisotropy, was developed in an earlier work [17]. It includes 52906 hexahedral elements and 100554 nodes. The Courtemanche-Ramirez-Nattel-Kneller membrane formalism [18], [19] was implemented to reproduce the human atrial cellular electrical activity. The monodomain model of the electrical propagation of the action potential along the tissue is described by a reaction-diffusion equation, and is solved using a finite element method [17].

To reproduce atrial electrical remodeling, changes in the maximum conductance and kinetics of different ionic channels of human atrial cells observed in experimental studies of chronic AF [20]–[22] have been incorporated into the atrial cellular model. The following parameters were altered [23]: the maximum conductance of 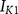 was increased by 100%, while the maximum conductance values of 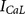 and 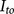 were decreased by 70% and by 50%, respectively.

#### Simulation protocol

Two AF episodes were generated by the S1–S2 protocol as follows: a train of stimuli with a basic cycle length of 1000 ms was applied for a period of 5 seconds in the sinus node area to simulate the sinus rhythm (S1). After the last beat of the sinus stimulus, a burst of 6 ectopic beats (S2) to high frequency were delivered into the right superior pulmonary vein, for the first AF episode; and they were delivered into the posterior wall of the left atrium near to the right pulmonary veins, for the second episode.

#### Virtual electrograms

Unipolar EGM in different points of the atria surface under conditions of uniform intracellular anisotropic resistivity was simulated, as previously described [24]. The extracellular potential (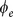) is given by the following equation:
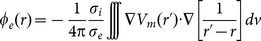
(1)where 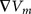 is the spatial gradient of transmembrane potential 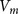, 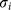 is the intracellular conductivity, 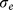 is the extracellular conductivity, 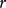 is the distance from the source point 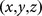 to the measuring point 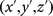 and 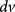 is the differential volume. EGM signals were recorded at 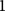 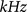. Bipolar EGM were calculated by subtracting two 1-mm-spaced adjacent unipolar EGM.

#### Numerical and computational methods

A hexahedral mesh was built from the three-dimensional anatomical model using Femap from Siemens PLM software. Equations were numerically solved using EMOS software [25]. EMOS is a parallel code (mpibased) that implements the finite element method and Operator Splitting for solving the monodomain model. The time step was fixed to 0.001 ms. Simulation of 10 seconds of atrial activity took 14 hours on a computing node with two 6-core Intel Xeon X5650 clocked at 2.66 GHz and 48GB DDR3 RAM.

### Isochrone activation maps

A method was developed as an arrangement of the isochrone map method [26], where the local activation times (LAT) are represented in a color map. To build an activation map, it is necessary to detect the local activation waves. An algorithm based on the Continuous Wavelet Transform was implemented to detect local activation waves. After a peak has been detected, an isochrone map is constructed with the LAT information. In order to ensure that one complete activation cycle is scanned, it is necessary to visualize the LAT minimum for a period of 100 ms. The points at which the activation waves are spinning can be observed; these points correspond to the rotor tip. This procedure was used as a gold standard for comparison with our results.

### Approximate entropy and parameter optimization

Approximate Entropy (ApEn) is a nonlinear statistic proposed by Pincus [27]. It quantifies the degree of complexity of signals. The calculation of ApEn depends on three parameters: number of data points 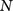, embedding dimension 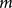 and threshold 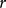. 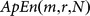 allows to measure regularity by calculating the probability that patterns of length 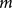 remain close on next incremental comparisons within a signal of length 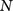, with 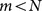 [28].

ApEn is theoretically defined as the value dependent on 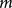 and 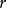, considering 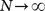. This value cannot be reached but can be approximated. The approximation is well suited when a significant number of patterns, determined by 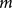, are acquired [29]. Pincus stated that small values of 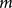 are needed in order to converge to the real value of ApEn [28]. Specifically, he suggested 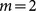, 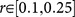 as standard parameters.

We evaluate 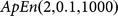 and 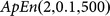 from standard parameters. Furthermore, we propose 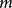 and 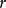 values derived from an optimization procedure using a dataset of EGM. This dataset has already been applied to other studies aimed at developing signal processing tools for CFAE [30], [31].

#### Dataset

We used two different EGM databases, independently recorded from AF patients, and independently evaluated. The databases were ranked by different electrophysiology teams, with different equipment, from two different countries. The 542 signals in the databases were classified using the same criteria divided into four classes: fractionated signals were categorized by experts into three levels of fractionation (C1, C2 and C3), and nonfractionated EGM signals were considered as level 0 (C0). The four fractionation classes, see Fig. 1B, are:

- C0: Nonfractionated EGM and also high frequency EGM.
- C1: Fractionated EGM with periodic activity.
- C2: A mixture of periodic fractionated and periodic nonfractionated EGM.
- C3: High frequency EGM with continuous activity. No regular activation can be seen.

The entire database constitutes a retrospective-offline analysis. For further information about the acquisition and classification of this database, refer to [32], [33]. The database is available at https://github.com/andresfod/Atrial_Electrograms_ApEn.

#### Optimization process

ApEn parameters 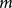 and 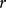 were set using real data from the database, to obtain optimal values. The variation of 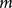 was limited to 5, as recommended by Pincus [27]. Parameter 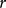 varied from 0.02 to 0.6, increasing in steps of 0.02. The database was organized into four levels of fractionation (C0, C1, C2 and C3). Each level contains the entire set of signals of the corresponding level of fractionation. The first 500 and 1000 ms of each EGM were considered. The numbers of EGM signals were as follows: 175 signals in C0, 117 signals in C1, 184 signals in C2, and 66 signals in C3. From now on, we refer to this database organization as DB-CZ-GE. For each combination of 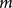 and 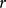, 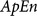 was calculated for each EGM from DB-CZ-GE and a boxplot was constructed, assigning a box to each level.

In the interest of minimizing the scatter of each class and maximizing the distances between the ApEn measures of the classes, two criteria were considered in the optimization procedure: interclass percentile distance 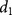 and interclass minimum-maximum distance 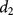, which were defined as follows:
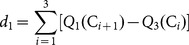
(2)

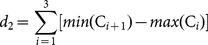
(3)where 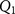 and 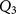 are the first and third quantile, respectively.

To select the parameters to be used for ApEn, an optimization function 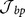 was constructed by equal weighting of criteria 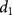 and 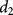 from (2) and (3):
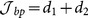
(4)


In order to validate the 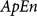 parameters obtained in the optimization procedure and to avoid overtraining, K-cross validation was performed. Randomly generated partitions were selected from full DB-CZ-GE. 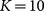 subsets were formed.

### Dynamic ApEn mapping

In order to generate a dynamic ApEn map over the surface of the atrial model, the standard ApEn parameters 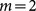 and 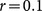, as suggested by Pincus [27], [28], [34] and the parameters of ApEn obtained in the optimization procedure, were used. ApEn was calculated for virtual unipolar EGM signals, using moving windows of 500 and 1000 points, without overlapping. The range of ApEn, over the entire set of virtual EGM, was applied to a color scale, where red color corresponds to 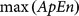 and blue color corresponds to 0. Each virtual EGM is related to an element in the model. A 1000-point window was applied for tracking stable rotors. A 500-point window was applied for tracking meandering rotors. Unipolar EGM over the entire surface of the atria were analyzed, with the exception of the meandering rotor case, in which an observation area was selected in order to evaluate the behavior of the optimized ApEn during the presence and absence of the tip of the rotor.

Bipolar-EGM based dynamic ApEn maps were also constructed for the stable rotor case. An observation area, in which the rotor tip is anchored, was selected. The ApEn values of bipolar EGM were calculated using the optimized parameters and the 1000-point window. Shannon Entropy (ShEn) maps were also generated, through a 4-second window and bins of 0.01 mV, in accordance with the work of Ganesan et al [7]. The ShEn performance in unipolar EGM was also assessed.

## Results

### CFAE mapping of stable rotors during AF simulation applying ApEn with standard parameters

**Figure 2:**
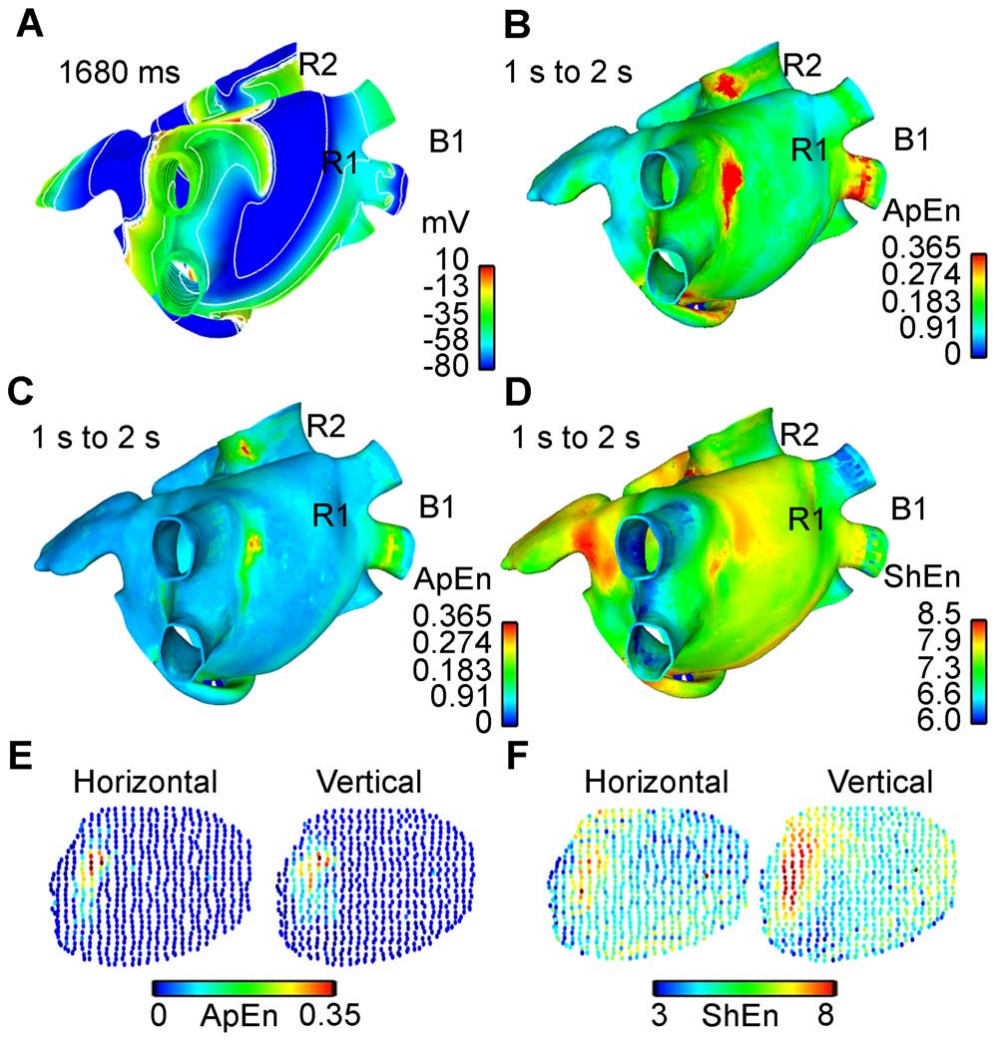
Comparison between tools for rotor mapping. A. Action potential wavefront delimited by contour lines over the 3D Human Atria Model extracted from the interval between 1 s and 2 s of simulation. The spinning wavefronts around one point define stable rotors R1 and R2. Line block B1 can be seen at the right inferior pulmonary vein. B. Dynamic ApEn map calculated using standard parameters and unipolar EGM. C. Dynamic ApEn map calculated from the optimized parameters obtained in our work, using unipolar EGM. D. Shannon entropy map, using unipolar EGM. Note that map C shows better sensitivity for localizing rotor tips. E. Dynamic ApEn map calculated from optimized parameters using bipolar EGM with horizontal and vertical orientation. The region corresponds to the vicinity of rotor R1. F. ShEn map calculated using the bipolar EGM obtained from the vicinity of rotor R1.

An AF propagation pattern over the atria was generated in the 3D model of human atria, see the action potential propagation in S1 Video. During AF activity initiated by an ectopic focus applied into the right superior pulmonary vein, two stable rotors were observed in the simulation. One is located in the posterior wall of the left atrium, near the left pulmonary vein, named R1, and the other is located in the superior vena cava, named R2. Fig. 2A shows the action potential wavefronts delimited by contour lines from the interval between 1 s and 2 s of AF simulation. Rotors R1 and R2, and a block line located over the inferior right pulmonary vein, named B1, have been marked. 42835 EGM were calculated in the whole atrial surface of the 3D model, over a four-second window.

For all EGM signals, the ApEn measurements were calculated using the standard parameters (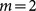, 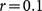). The ApEn values were used to generate a color map over the anatomic structure of the atria in a 3D model. In this manner, a dynamic ApEn map was obtained and the frame corresponding to the time interval from 1 s to 2 s is depicted in Fig. 2B. Red color areas, corresponding to high ApEn values (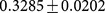), include B1 and the coronary sinus (CS) and also R1 and R2.

### Optimization of ApEn parameters (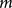 and 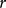)

**Figure 3:**
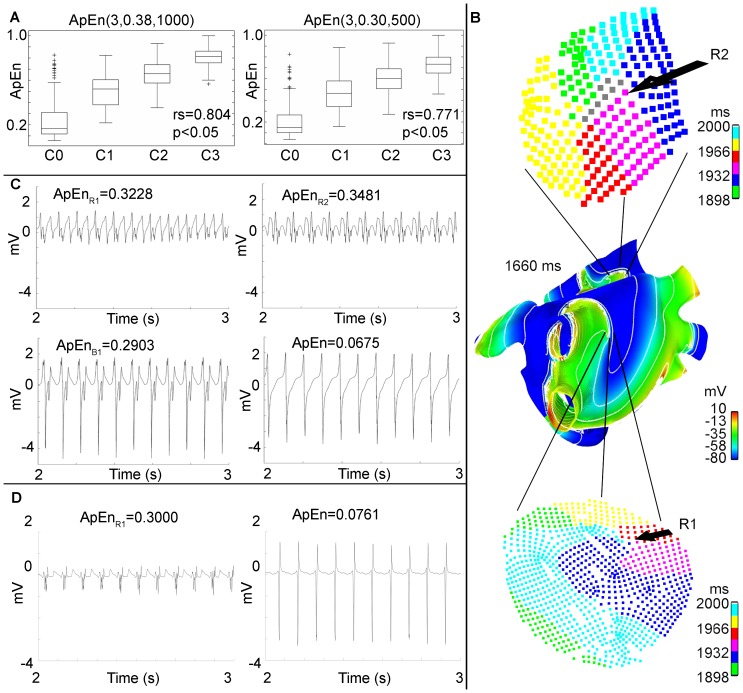
Localization of stable rotors. A: Results of the optimization procedure. Boxplots of ApEn normalized values using optimized parameters: 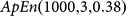 (left) and 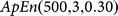 (right). The Spearman correlation coefficient calculated over DB-CZ-GE for each boxplot is shown. B: Activation isochronic maps corresponding to R1 (below) and R2 (top). The rotor tip is indicated where the colors converge. C: EGM generated by the model in the areas of stable rotors and the block for the time interval between 2 s and 3 s. EGM corresponding to the R1, R2, B1 and plane activation wavefront areas. D: Bipolar EGM corresponding to R1 and plane activation wavefront area. ApEn values for each EGM are shown.

The optimization procedure resulted in 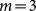, 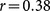, for 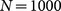 and 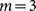, 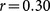, for 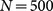. Fig. 3A shows the boxplots for 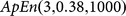 and for 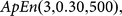 respectively, applied to DB-CZ-GE. In addition, the Spearman correlation coefficient 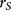 between ApEn and the corresponding level of fractionation was calculated.

### CFAE mapping of stable rotors during AF simulation applying ApEn with optimized parameters

A dynamic 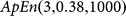 map was generated using unipolar EGM, and the frame corresponding to the time interval 1 s to 2 s is depicted in Fig. 2C, see S1 Video. The dynamic ApEn map reveals two areas of high ApEn values (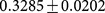), corresponding to rotors R1 and R2. In addition, the third area with lower ApEn values (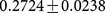) corresponds to B1, taking into account that passive activation regions have ApEn values lower than 0.1. A ShEn map was also generated and is shown in Fig. 2D. Red color areas, corresponding to high ShEn values (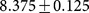), include small vicinities near to R1 and near to B1, along other zones, e.g. CS, the inferior wall of the left atria, the left and right appendage and the lateral wall near to the left appendage. The LAT method was applied as a reference method, see S2 Video. Fig. 3B shows isochronic maps from R1 and R2. The rotor tip is defined by the converging points of the colored waves. These waves represent the local activation. Green color indicates early activation points. Table 1 shows the spatial location of R1 and R2 found using the LAT method and the dynamic ApEn mapping method. The last column corresponds to the Euclidean distance between the spatial coordinates from the rotor tip, identified using both methods. The maximum distance is 1.66 mm. Unipolar EGM extracted from rotor sites R1 and R2, and near to block area B1, are shown in Fig. 3C. ApEn values are also presented. The EGM corresponding to rotor activity (top) have low voltage and irregular morphology. The EGM corresponding to the block line (below left) presents fractionation, but activation patterns are visible and their amplitudes are similar to non-fractionated EGM (below right). The highest ApEn values correspond to R1 and R2.

**Table 1:** Spatial rotor localization.

	Coordinates			Coordinates			Euclidean
	LAT method (mm)			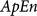 method (mm)			distance (mm)
Rotor tip R1							
Interval (s)	x	y	z	x	y	z	d
0 to 1	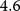	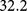	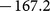	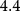	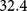	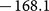	0.91
1 to 2	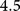	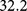	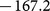	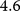	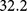	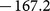	0.00
2 to 3	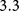	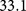	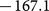	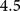	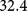	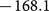	1.66
3 to 4	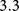	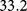	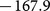	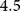	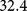	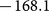	1.44
Rotor tip R2							
Interval (s)	x	y	z	x	y	z	d
0 to 1	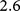	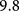	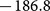	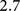	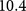	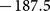	0.95
1 to 2	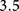	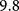	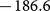	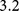	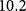	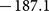	0.66
2 to 3	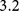	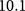	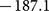	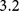	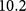	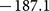	0
3 to 4	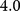	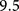	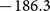	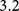	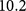	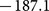	1.32

Comparison between ApEn and the LAT method. Spatial location of 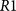 over the 3D atria anatomical structure. The coordinates were found by two methods: the LAT method, as a reference, and the 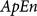 map method. Euclidean distance is used to compare the performance of the two methods for four time intervals 1 s in length.

A circular area containing R1 was selected. Bipolar EGM were calculated using horizontal and vertical bipole orientation. 636 EGM were obtained for each orientation. Fig. 2E shows the dynamic 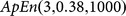 maps for horizontal and vertical bipolar orientation, in the time interval between seconds 1 and 2. High ApEn values correspond with the tip of the rotor in both cases. Fig. 2F shows the ShEn maps. The vertical bipolar orientation map presents a region of high ShEn values broader than the horizontal orientation map, though both contain the tip of the rotor. Bipolar EGM and its ApEn values are shown in Fig. 3D. The EGM corresponding to R1 (left) have low voltage and an irregular morphology. The EGM corresponding to a region outside the rotor tip (right) presents high amplitude, high frequency activation patterns and a regular morphology.

### CFAE mapping of a meandering rotor during AF simulation applying ApEn with optimized parameters

**Figure 4:**
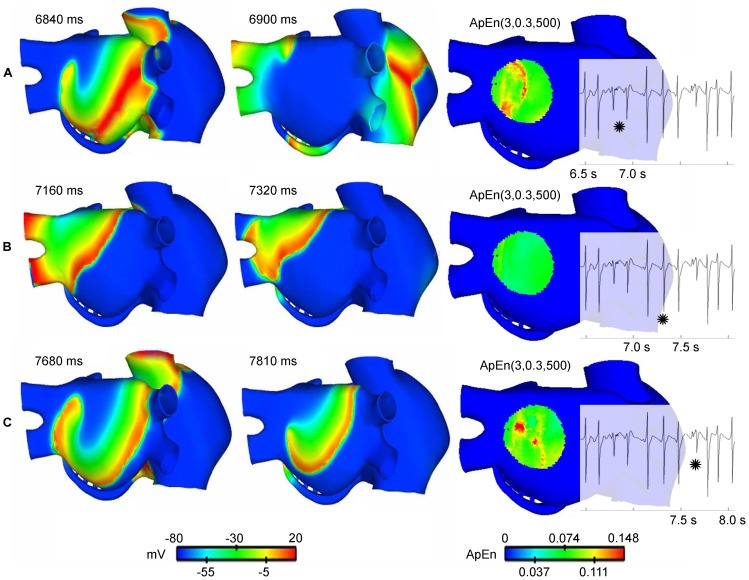
Meandering rotor tracking. The left and centre snapshots in A, B and C show the evolution of the action potential at three time instants. Meandering rotational activity is present in A and C. The snapshot on the right corresponds to the dynamic ApEn(3,0.30,500) maps. High ApEn values (red color) correspond to the presence of a meandering rotor. EGM is also shown. The star marks the 500-point interval corresponding to the evolution of the action potential. Fragmentation is generated in the presence of a rotor (A and C).

During AF activity initiated by an ectopic focus applied in the posterior wall of the left atrium near to the right pulmonary veins, a meandering rotor was observed in the posterior wall, see the action potential propagation in S3 Video. 933 unipolar EGM signals of 9 seconds length were obtained from the AF simulation. The ApEn measurement was calculated using the parameters found to be optimal (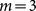, 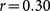). The ApEn values were used to generate a color map in a 3D model, see S3 Video. The left and centre maps in Fig. 4 show the evolution of the action potential during a time interval when the rotor tip meanders. Figs. 4A and 4C show the rotor activity, while Fig. 4B shows an action potential map with a plane wavefront over the zone. The dynamic ApEn(3, 0.30, 500) maps shown on the right side of Fig. 4 present high ApEn values only in intervals corresponding to Figs. 4A and 4C. An EGM localized over the area where the rotor meanders is also shown; fractionation of the EGM is observed when the rotor is in the zone.

## Discussion

The principal findings of our work can be summarized as follows:

- ApEn values with optimal parameters are strongly and positively correlated with the fractionation levels defined in the AF EGM database.
- High ApEn values were found in EGM from stable and meandering rotor tips in a simulated episode of AF.
- We have developed a methodology for localizing the tip of the rotor during AF, using dynamic ApEn maps.

### Dynamic ApEn maps for detecting rotor tip sites

A method based on dynamic ApEn maps is presented in this study as a basis for proposing a new tool for automatic detection of ablation target sites. We have presented evidence that the method is able to detect rotors during two simulated AF episodes. We used the continuous ApEn value for building color maps. Fig. 2C shows the localization of the stable rotors, and Fig. 4 shows the localization of the meandering rotor. The red areas in the ApEn map correspond to the location of rotor tips. This is an indicator that high ApEn values are related with rotor activity and, more precisely, with rotor tips. The mechanism behind the unipolar fractionation in our simulations can be explained as follows: when the rotor turns around the pivot point (tip), the tip is affected by wavefronts from the rotor head. When the wavefront passes near to the tip in each rotation cycle, several electrotonic potentials are observed, consequently, fractionation arises and there is irregularity. The ApEn response to the occurrence of such fractionation is as follows: if the rotor is stable, the unipolar EGM, registered at the tip, will present fractionation as long as the rotor tip remains harbored to that point. This causes ApEn to increase over the entire observing window. If the rotor meanders, fractionated complexes will be observed when the rotor tip passes over that specific point, therefore increments of the ApEn will be expected in that instant of time. Our results are consistent with other works, in which fractionated unipolar EGM were observed at pivot points [35] and unipolar EGM symmetry is affected by the wavefront curvature [36]. Moreover, the action potential map, shown in Fig. 2A, indicates a lobule B1 that does not depolarize when a wavefront arrives. This irregular activity increases the ApEn value but not to rotor levels, as is shown in Fig. 3C. This ApEn level corresponds to the yellow areas. Umapathy et al [11] reported that CFAE were located in sites of wave breaks and in the region of a rotor tip during experiments in a murine HL-1 atrial monolayer model. Their results agree with ours in the 3D model. Furthermore, we show the ability of dynamic ApEn maps to locate and to distinguish between these substrates.

We found that high ApEn values are related with arrhythmogenic substrates, such as rotors and block lines. Discrimination between active and passive CFAE is still an open question [5]. One important question is how to differentiate CFAE according to the substrate that generates it. Using 2D computer models and cell cultures, Navoret et al detected CFAE using amplitude criteria, number of deflections and cycle length. They established a relationship between the detected CFAE and the presence of shock waves and rotors, but they failed to differentiate them [37]. Another study reported an algorithm that classifies CFAE and non-CFAE, but fails to distinguish between active CFAE (whose ablation restored the sinus rhythm) and passive CFAE (whose ablation did not restore the sinus rhythm) [8]. Our study showed that the dynamic ApEn map, calculated using standard ApEn parameters (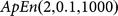), identifies R1, R2, B1 and CS having high ApEn values, however it is not possible to differentiate between them. On the other hand, the ApEn dynamic map, calculated with optimized parameters ApEn(3,0.38,1000), assigns scaled values to zones of interest: rotors R1 and R2 have the highest ApEn value, followed by intermediate ApEn values in block line area B1 and the wave collision in the CS, and lower ApEn values for fibrillatory EGM with an organized activity. These results suggests that the optimization process for ApEn improves the detection and discrimination between rotor tips, block-lines and wave collisions, and this could help in solving the question discussed above.

### Variability and irregularity of fractionated EGM during AF

We hypothesized that the AF EGM has variability at the tip of the rotor, but we also expected irregularity (e.g. fractionation of the EGM). So we used ApEn to quantify the variability and the irregularity at the same time in the following manner: ApEn was calculated from segments of equal length but shorter than the entire signal. Thus, we ensure a quantification of the variability by calculating time consecutive measures of regularity. That is why the time series information aspect is important. However, time series information by itself is not enough to localize rotors. Spatial information (i.e. the spatial coordinates of each EGM) allows a rotor to be situated over the atria.

This study has taken into consideration the morphological features of both unipolar and bipolar EGM under two provisos: 1) Fractionation has been described in unipolar and bipolar EGM [35], [38]. 2) Fractionated EGM have an irregular morphology. We found that ApEn is related with the degree of fractionation: ApEn values tend to be higher if the degree of fragmentation is higher. Therefore, the use of bipolar signals and unipolar signals does not affect the performance of ApEn. This is evidenced in the results for the location of the rotors using unipolar and bipolar EGM in the computational model using ApEn optimized with the bipolar EGM of the multicenter DB-CZ-GE.

ApEn was developed as a measure of regularity to quantify levels of complexity within time series [39], [40]. Since we hypothesized that fractionation of EGM could be graded, with the highest level at the tip of the rotor, we chose ApEn to quantify levels of fractionation, as this is a well-characterized measure in other cardiac applications, e.g. RR intervals, heart rate variability signals and ECG signals [41]. Hoekstra et al [42] showed that the complexity of EGM increases with the type of fibrillation, based on the chaotic spatio-temporal activation patterns of the right atria. In a study on animal models, Ganesan et al [7] found that there are some similarities in the visual appearance of EGM signals with higher ShEn and fractionated signals. Novak et al [43] compared various measures from the theory of nonlinear dynamics that can help to provide an objective description of the level of fractionation and the complexity of CFAE signals. Thus, non-linear tools can provide useful information about the occurrence of arrhythmogenic substrates.

Figs. 3C and 3D show four unipolar and 2 bipolar EGM morphologies observed in the computational model, respectively. The EGM from rotors (top in Fig. 3C and left in Fig. 3D) have low voltage and irregular morphology whereas that, the EGM from sites with a plain wavefront (bottom in Fig. 3C and right in Fig. 3D) are regular. Furthermore, the ApEn values graded the bipolar EGM from DB-CZ-GE according to the level of fractionation (Fig. 3A). Again, the increasing irregularity in the four fractionation classes in DB-CZ-GE can be visually verified in Fig. 1B. Thus, we demonstrate that ApEn is well correlated with the perception of morphological regularity of EGM. This feature of ApEn was already reported in the work of Anier et al [44], though in electroencephalograms. Additionally, we found that there are different fractionated unipolar EGM morphologies or levels of fractionation, depending on the wave propagation pattern in the 3D model.

### Other rotor detection tools and fractionation measures

We developed the dynamic ApEn maps tool considering fractionation of EGM as non-linear dynamics and considering that degrees of complexity can be identified within fractionated EGM. To the best of our knowledge, no other tools have been developed that take these two features into account. However, there are other similar studies regarding rotor detection. Ganesan et al [7] were able to relate high ShEn values with the tip of the rotor in 2D arrays using bipolar EGM and the directional information they contain. We were able to reproduce these results using bipolar signals from the 3D model corresponding to a zone that encloses R1 (Fig. 2F). We have also applied optimized ApEn to the bipolar EGM, successfully detecting the tip of R1 (Fig. 2E). Both approaches define an area enclosing the rotor tip, where the ShEn values decrease more slowly than ApEn in the area surrounding the tip. Thus, ApEn gains in specifying the tip of the rotor in our simulations. Furthermore, ApEn offers reduced dependence on the orientation of the record. We assessed the ShEn map using unipolar EGM (Fig. 2D), in which substrate detection was not accomplished. These results suggest that dynamic ApEn maps could be used in unipolar and bipolar EGM for the task of rotor mapping.

Narayan et al [26] used focal impulse and rotor modulation to guide ablation with better results than when using ablation without this approach. They used the LAT method to localize the rotor tip. We compared the dynamic ApEn maps with the LAT method, and obtained differences less than 2 mm. The use of isochronic activation maps is limited by the high computational cost for constructing the maps, and the fact that they need to observe several frames in order to study transient rotational waves – a procedure that is likely to take considerable time. This implies that meandering rotors are hard to detect using isochronic activation maps. Dynamic ApEn maps can detect a stable rotor, see S1 Video, where the red sites do not change over time, or they can track a meandering rotor tip, see S2 Video, where the red sites change over time. This technique can help to reduce the uncertainty in the location of the rotor from the activation maps.

Zlochiver et al [12] studied the regularity of EGM in the presence of stable rotors. Spectral analysis was applied to the singular value decomposition in order to measure the contribution of the meandering frequency and the rotor tip frequency. They also assessed regularity using the periodicity index. Rotor tips were detected in 2D computational arrays, through low periodicity index values calculated from unipolar EGM. Fractionation of the EGM was observed at the rotor tip. These results support and agree with ours, though the two works apply different approaches: Zlochiver et al exploit the fact that under fractionation conditions, the periodicity of the signal is lost, which can be observed in the singular components. We consider fractionation as a non-linear phenomenon, and we asses the morphological features of the signal using ApEn. Although the work of Zlochiver et al reports the possibility of detecting stable rotors, it would be interesting to asses whether the tool that they have developed would work with meandering rotors, as ours does. We can see that they need a signal of 2000 seconds, which would initially limit the temporal resolution to tracking a meandering rotor (we used a 500-point window).

Existing measurements of CFAE, implemented in electroanatomical mapping devices, are based on Nademanee's definition of CFAE. There are studies demonstrating that CFAE are not suitable for ablation of AF. Narayan et al [16] graded CFAE applying the interval confidence level (ICL) algorithm [45] which is implemented in Carto XP. They conclude that most CFAE are not able to localize AF sources. Ganesan et al [7] compared the ShEn results with CFAE analysis (CFE-mean) performed in NavX. They observed a consistent but weak inverse correlation between ShEn and CFE-mean. It is worth pointing out that both ICL and CFE-mean algorithms use cycle length and voltage amplitude criteria under the definition of CFAE given by Nademanee [3]. They are sensitive to changes in the morphology of the signal, which can have different appearances depending on the contact between the catheter and the substrate [10], or can be influenced by far-field artifacts or by noise [46]. Our results suggest that rotors can be localized by considering different levels of fractionation and non-linear tools. These observations invite to enhancing the Nademanee's definition of CFAE. His definition is based on cycle length and amplitude criteria, which are not sufficient for describing the levels of fractionation. It may be better to consider defining CFAE as a nonlinear dynamic phenomenon. This last statement is supported by other authors [4], [6], [47], [48]. It could be more effective to consider fractionation as a complex phenomenon, the description of which should be resolved from various fractionation levels, rather than using a CFAE/non-CFAE classification.

Although the remodeling conditions that are used can reproduce the action potential phenotype observed in patients with permanent AF, electrical remodeling is not the only process accompanying chronic AF. Indeed, many of these patients have significant structural remodeling with fibrosis, which contributes to short APD and increases the complexity of the arrhythmia. The 3D anatomical model of human atria does not take into account the real thickness of the atrial walls. Our results were obtained using a specific virtual atria model. Although our model includes a great number of anatomical and morphological details, it corresponds to a particular set of parameters (electrophysiology, anatomy, fiber direction, anisotropy and heterogeneity, among others). In addition, although there are also inter-subject differences in fiber orientation, we have tried to model the most common fiber orientation observed experimentally for the different parts of the atrial model. Regarding EGM analysis, although we assessed the performance of ApEn and ShEn using bipolar EGM, it was executed over a small area due to the high complexity of defining a bipolar record configuration over the whole atria. The behavior of both ApEn and ShEn, calculated using bipolar EGM over broader regions, including other arrhythmogenic mechanisms besides rotors, should be studied. In order to fully validate the results of our optimization procedure with DB-CZ-GE, although good performance has been achieved for rotor detection in the 3D model, future studies should involve collecting more AF EGM samples from other databases in order to achieve equally distributed fractionation levels. Additionally, real unipolar EGM must be included.

In conclusion, we have provided evidence for postulating dynamic ApEn maps as a supporting tool in AF ablation procedures. ApEn calculation over atrial EGM signals can be used to build an electroanatomical map with continuous ApEn values represented in colors that can identify rotor tip locations without pre-specifying thresholds. This property makes the method adaptable to specific electrophysiological cases. A combination of all this could help to improve ablation procedures. We suggest that dynamic ApEn color maps could become a tool for AF rotor mapping. The methodology proposed here needs to be validated by experimental models and by clinical studies to evaluate ablation procedures guided by dynamic ApEn maps.

## Supporting Information

S1 Video **Stable rotors.** Left: Simulation of an AF episode induced by six transitory ectopic beats applied in the ostium of the right pulmonary vein, sustained by multiple reentrant waves. Two rotors can be observed: in the posterior wall of the left atria posterior wall, and in the superior cava vein. Right: A dynamic ApEn map using optimized parameters. Red areas correspond to the tips of rotors R1 and R2.
(MP4)

S2 Video **Isochrone map.** Isochrone maps for rotors R1 and R2 for the time interval between 1500 ms and 2000 ms.
(MP4)

S3 Video **Meandering rotor.** Left: Simulation of an AF episode induced by six transitory ectopic beats applied in the posterior wall of the left atrium near to the right pulmonary veins, sustained by multiple reentrant waves. One meandering rotor can be observed in the posterior wall of the left atria. Right: A dynamic ApEn map using optimized parameters. Note that, when a plane activation wavefront is present within the observation area, the dynamic ApEn map shows low values (green). Red zones appear when the rotor tip is within the observation area (e.g. the time interval between 2500 ms and 3000 ms).
(MP4)
