# Correction: Dynamic Approximate Entropy Electroanatomic Maps Detect Rotors in a Simulated Atrial Fibrillation Model

**DOI:** 10.1371/journal.pone.0118677

**Published:** 2015-03-05

**Authors:** The PLOS ONE Staff

The affiliation for the fifth author is incorrect. Daniel Novak is not affiliated with #3 but with #4 Department of Cybernetics, Faculty of Electrical Engineering, Czech Technical University in Prague, Prague, Czech Republic.
